# The roles of health literacy in parents’ honey use and the use of complementary alternative medicine in a Turkish population

**DOI:** 10.1186/s12906-023-04209-z

**Published:** 2023-10-23

**Authors:** Suzan Tek Ayaz

**Affiliations:** Yozgat Bozok University, Akdagmadeni School of Health, Nursing Department, Yozgat, Turkey

**Keywords:** Parents, Health literacy, Complementary and alternative medicine, Honey

## Abstract

**Background:**

As a biologically based therapy, honey is used by parents in many parts of the world as a home remedy for their children. While information exists regarding the traditional use of honey for health issues in children, data regarding its relationship with health literacy is lacking. The aims of this study were to determine the use of honey to address children’s health issues among parents of children aged 0–72 months and to explore the relationship between the use of complementary and alternative medicine (CAM) and health literacy.

**Methods:**

The data for this descriptive, cross-sectional study were collected between October and November 2022 via an online survey of 907 parents of children aged 0–72 months. A sociodemographic data collection form, the Holistic Complementary and Alternative Medicine Questionnaire (HCAMQ), and the Turkey Health Literacy Scale-32 (THLS-32) were used to collect the data. A t-test, one-way analysis of variance, and Pearson correlation analysis were used to analyze the data.

**Results:**

The majority (86.5%) of the parents used honey for their children’s health problems. Among the parents, 83.1% utilized honey as a remedy for alleviating cough symptoms, 10.4% employed it as a treatment for diarrhea, and 14% administered it for the management of oral mucositis. The mean THLS-32 and HCAMQ scores of the parents were 29.39 and 21.31, respectively, and there was a moderate correlation between the parents’ THLS-32 and HCAMQ mean scores (r = 0.662, p < 0.001).

**Conclusion:**

This study revealed that a significant proportion of parents who used honey to alleviate their children’s health issues displayed positive attitudes toward complementary and alternative medicine (CAM) while concurrently exhibiting insufficient or limited health literacy levels. Therefore, it is advisable to enhance health literacy regarding the proper and safe utilization of honey, which functions as a biologically based CAM therapy.

## Background

Eighty-eight per cent of the world’s population uses complementary and alternative medicine (CAM) therapies [1]. This percentage may be even higher in cases related to child health because parents often resort to traditional and complementary approaches to protect their children’s health and provide care during times of illness [2]. While the global incidence of CAM utilization among pediatric populations exhibits a wide range, spanning from 10.9 to 93.3% [3], a systematic review conducted in the context of Turkish pediatric patients revealed a notably high frequency of CAM adoption, reaching 87% [2].

The National Centre for Complementary and Alternative Medicine has classified CAM modalities into five broad groups: alternative medical systems, or complete systems of therapy and practice; mind-body interventions, or techniques; manipulative and body-based methods; energy therapies; and biologically-based therapies [4]. Studies have reported that honey is a functional food in the most commonly used biological-based therapy group [5]. In many countries such as Ghana, Indonesia, Bangladesh, Argentina, the United States, Italy, Malaysia and Turkey, honey has been reported to be used as CAM [5–12]. Honey has traditionally been used by parents for the treatment of problems such as cough, asthma, allergic rhinitis, and oral thrush for a long time. While accessible data are available regarding the frequency of parents’ honey use, research findings in the field of CAM indicate that parents use honey either alone or in combination with other medicinal herbs [5, 8–12]. Scientific evidence has substantiated that honey, traditionally utilized for thousands of years, possesses properties such as an acidic pH, high osmolarity, and low water content, as well as antibacterial and antioxidant therapeutic attributes [13, 14]. Health authorities and recent scientific studies suggest using honey (except for infants under one-year-old due to the risk of infant botulism) as an alternative remedy for ailments including coughs, sore throats, burns, infected wounds, skin ulcers, diarrhea, and oral mucosa inflammation caused by chemotherapy/radiotherapy [15–18]. On the other hand, researchers also underscore the necessity for caution in honey consumption, owing to the potential risk of contamination with botulinum spores, pesticides, heavy metals, and radioactive substances [19]. Therefore, parents should possess knowledge and awareness concerning the use of honey, be it within the framework of traditional usage or guided by research-based information.

In society, the utilization of complementary and alternative medical therapies, such as honey, is prevalent. However, it has been reported that individuals possess inadequate knowledge about their benefits, risks, and side effects, with a significant portion of them obtaining information from unreliable sources [20] The significance of parental health literacy levels has been underscored in order to be knowledgeable about CAM therapies, employ information effectively, and distinguish the reliability of information concerning these methods [20]. The existing body of research that has investigated the relationship between health literacy level and CAM utilization has yielded inconsistent results. Several studies contend that there is no apparent correlation between health literacy levels and CAM utilization, whereas another investigation found that an increase in health literacy levels was associated with a decrease in positive attitudes towards CAM [21–23]. Yet, one other study highlighted the association between adequate or high health literacy and CAM use [24]. There is a notable gap in the available data regarding the relationship between individuals who use honey as one of the CAM therapies and their CAM attitudes and health literacy levels. Considering the high prevalence of CAM usage, including honey, among parents in Turkey for the purpose of preserving their children’s health, alleviating symptoms, and achieving positive health outcomes, and recognizing that approximately seventy per cent of individuals in Turkey have a low health literacy level, it becomes imperative and vital to elucidate the relationship between parents’ CAM attitudes and their health literacy levels [25]. Considering the aforementioned context, the objectives of this study were to investigate the utilization of honey by the parents of children aged 0–72 months to address their children’s health issues and to define the relationship between health literacy and the utilization of CAM.

## Methods

### Study design and sample

The data for this descriptive, cross-sectional study were collected in October and November 2022. According to the data obtained from the Yozgat Health Directorate and the Turkish Statistical Institute, the total number of children aged 0–72 months in the district where the research was conducted was 7892 [26, 27].

In the calculation of the sample size, the formula n = z^2^P(1-P)/d^2^ was utilized. This revealed that using parameters of power = 80%, Z = 1.96, d = 0.05, and p and q = 0.5, the minimum required number of parents to be included in the sample should be 384. A self-administered online questionnaire disseminated over different platforms, including Email, Telegram, Twitter, WhatsApp, Instagram, and Facebook, through a Google Doc URL link was used to collect data. Considering that web surveys generally have a lower response rate compared to other survey modes, the survey was shared at least twice on each of several social media platforms to reach parents of children aged 0–72 months in district [28]. When sharing the Google Docs URL on social media platforms, a brief invitation description was included. This brief communication invited parents residing in the district where the research was conducted and who used honey for their young children’s health issues, with children aged 0–72 months, to participate in the study. Prior to addressing the research questions, an informed consent form containing information about the purpose of the study and the right to withdraw from the study was presented to parents through an online survey Google Doc URL link. They were asked to mark the statement “I agree to participate in the study” if they agreed to participate. An email address checked daily, was provided for participants to seek further information on the study if needed. Multiple interactions were conducted with numerous parents, addressing the questions they posed. Out of the 1002 parents who read the informed consent form and explicitly indicated their willingness to participate, 907 parents who provided complete responses to the questions were included in the study. The complete response rate was 90.5%. The inclusion criteria for the study were as follows: being 18 years or older, having children aged 0–72 months, having access to the internet, being able to use a smartphone/tablet/computer, living in the district where the research was conducted, and agreeing to participate in the study.

This study was conducted in accordance with the Declaration of Helsinki, and the Institutional Review Board of Yozgat Bozok University approved the research protocol (approval number: 40/02, date: 11/21/2022). Informed consent was obtained from all participants in this study. The responses of the parents who participated in the study were treated as confidential and only accessible to the researcher responsible for account credentials.

### Data collection instruments

The data were collected using the data collection form developed based on the literature, the Holistic Complementary and Alternative Medicine Questionnaire (HCAMQ), and the Turkey Health Literacy Scale-32 (THL-32).

#### Data collection form

In line with the relevant literature, a data collection form tailored to the research objectives was devised [8, 12]. The data collection form consisted of two sections, one dedicated to capturing respondents’ sociodemographic details and the other focused on their patterns of honey use. In the sociodemographic characteristics section of the data collection form, six questions were included, covering their child’s age and gender, as well as the parents’ age, gender, education, and household income. In the second section of the data collection form, there were eight questions related to parents’ use of honey in the past year for their children’s health concerns. These questions covered the reasons for choosing honey, the source of obtaining honey, their knowledge of honey content, sources of information, knowledge of botulism, and its use for addressing their children’s health issues such as cough, diarrhea, oral mucositis, etc.

#### HCAMQ

The HCAMQ was developed by Hyland et al. (2003), and Erci (2007) determined its validity and reliability in Turkey [29, 30]. The scale consists of 11 six-point Likert-type items (1 = strongly agree, 6 = strongly disagree) in two subdimensions. Six of the HCAMQ items relate to attitude about the CAM subdimension (items 2, 4, 6, 8, 9, and 11), and five to beliefs about holistic health (items 1, 3, 5, 7, and 10). Items 2, 4, 6, and 9 in the scale are scored in reverse. The lowest score that can be obtained from the scale is 11, and the highest score is 66. A low score on the scale indicates a positive attitude toward CAM, and a high score indicates a negative attitude [29, 30]. Cronbach’s alpha for the scale was 0.72, and the value for this study was calculated as 0.70.

#### THLS-32

The THL-32 is a 32-item scale developed based on the conceptual framework of the European Health Literacy Survey by Okyay et al. [31, 32]. The scale consists of 32 five-point Likert-type items (1 = very easy, 2 = easy, 3 = difficult, 4 = very difficult, 5 = I have no idea). In the evaluation of the scale, the indexes are standardized to fall within the range of 0 to 50 points, as in the HLS-EU study [31]. For this purpose, the formula [index = (mean − 1) x (50/3)] is employed. Zero points indicate the lowest level of health literacy, while 50 points represent the highest level of health literacy. Specifically, scores ranging from 0 to 25 points indicate insufficient health literacy, 26 to 33 points signify problematic-limited health literacy, 34 to 42 points indicate adequate health literacy, and scores falling between 43 and 50 points reflect excellent health literacy. The scale demonstrated an overall internal consistency coefficient of 0.92. In this particular study, the Cronbach’s alpha value for the scale was determined to be 0.95 [31, 32].

### Data analysis

The data were analyzed using the IBM Statistical Program for Social Sciences version 22.0. The demographic data of the respondents was analyzed using descriptive statistics. The normal distribution of the data was tested for kurtosis and skewness values (HCAMQ kurtosis: −0.744, skewness: −0.342; THLS-32 kurtosis: 0.410, skewness: −0.174). Kurtosis and skewness values between − 1.5 and + 1.5 indicate that the data are normally distributed [33]. The data was subjected to analysis using a t-test, one-way analysis of variance, and Pearson correlation analysis. A p-value less than 0.05 was accepted as statistically significant.

## Results

The mean ages of the children and their parents were 48.27 ± 9.8 months and 39.6 ± 8.7 years, and 53.7% and 55.6% were female, respectively. Among the parents, 48.4% were high school graduates, and 56.2% had an income equal to their expenses. The reasons for their choice of honey were its accessibility (49.6%), perceived high efficacy (34.8%), and absence of side effects (15.5%) compared to conventional medicine.

Of those parents who participated in the survey, 93.7% expressed a preference for natural honey. Furthermore, the survey revealed that 95.4% of the respondents lacked knowledge regarding the content of honey, while 45.9% relied on friends and family as their primary source of information on honey.

The rate of parental honey consumption before their child’s first year was 12.2%, and only 4.3% were knowledgeable about honey’s potential to cause botulism. The proportion of parents using honey to address their children’s health issues was 86.5%. Among them, 83.1% used honey to alleviate coughs, 10.4% for diarrhea, and 14% for oral mucositis (Table 1).

**Table 1:** Sociodemographic and honey use characteristics of participants with mean scores for THLS-32 and HCAMQ

Sociodemographic characteristics			THLS-32		HCAMQ	
	N	%	Mean (SD)	Min–max	Mean (SD)	Min–max
Child						
Age: 48.27 ± 9.89 months (8–72 months)						
0–12 months	87	9.6	29.49 (7.26)	25–45	21.62 (5.39)	14–39
13–36 months	372	41.0	29.31 (7.13)	25–47	21.23 (4.43)	11–38
37–72 months	448	49.4	29.37 (7.62)	19–25	21.44 (2.79)	12–21
	F = 0.248, p = 0.766			T = 0.065, p = 0.937		
Gender						
Female	487	53.7	29.30 (7.20)	16–47	21.37 (6.13)	11–39
Male	420	46.3	29.49 (7.32)	16–47	21.25 (6.14)	11–39
	T = 0.154, p = 0.881			T = 0.204, p = 0.827		
Parents						
Age: 39.65 + 8.79 years (24–63 years)						
24–34	311	34.3	35.05 (4.67)	25–45	26.19 (5.39)	14–39
35–44	264	29.1	31.86 (6.07)	25–47	22.20 (4.43)	11–38
45–54	299	33.0	22.64 (1.96)	19–25	16.54 (2.79)	12–21
55 and over	33	3.6	17.33 (0.88)	16–19	11.51 (0.50)	11–12
	F = 19.54, p < 0.001			F = 18.30, p < 0.001		
Gender						
Female	504	55.6	29.35 (7.19)	16–47	21.35 (6.15)	11–39
Male	403	44.4	29.43 (7.33)	16–47	21.27 (6.11)	11–39
	T = 0.155, p = 0.877			T = 0.205, p = 0.837		
Education						
Primary/secondary school	328	36.2	22.08 (2.45)	16–25	15.98 (3.02)	11–21
High school	439	48.4	30.99 (3.56)	25–38	24.13 (5.70)	11–39
University	140	15.4	41.49 (2.62)	38–47	24.98 (3.94)	20–36
	F = 9.40, p < 0.001			F = 21.48, p < 0.001		
Household income						
Income less than expenses	237	26.1	29.10 (7.26)	16–47	21.21 (6.12)	11–39
Income equals expenses	510	56.2	29.51 (7.13)	16–46	21.37 (6.11)	11–39
Income more than expenses	160	17.6	29.43 (7.62)	16–47	21.28 (6.25)	11–38
	F = 0.259, p = 0.772			T = 0.060, p = 0.942		
Honey use characteristics						
Reasons for honey choice						
Accessible	450	49.6	27.37 (6.90)	16–47	19.12 (6.05)	11–39
Effective	316	34.8	32.63 (6.52)	18–46	24.95 (4.77)	12–39
No side effects	141	15.5	28.57 (7.38)	16–46	20.17 (5.29)	11–30
	F = 46.91, p < 0.001			F = 30.51, p < 0.001		
Source of obtainment of honey						
Natural	850	93.7	29.35 (7.19)	16–47	21.29 (6.12)	11–39
Processed	57	6.3	29.89 (8.07)	17–47	21.63 (6.38)	11–37
	T = 0.540, p = 0.589			T = 0.396, p = 0.692		
Honey content knowledge						
Yes	42	4.6	45.00 (0.88)	43–47	22.88 (3.45)	20–36
No	865	95.4	28.63 (6.53)	16–43	21.24 (6.22)	11–39
	T = 16.21, p < 0.001			T = 1.69, p = 0.091		
Sources of information						
Family, friends	416	45.9	29.49 (7.13)	16–47	21.29 (6.11)	11–39
Media	252	27.8	29.44 (7.62)	16–46	21.28 (6.25)	11–39
Social media	239	26.3	29.11 (7.26)	16–47	21.19 (6.12)	11–39
	F = 0.258, p = 0.771			T = 0.062, p = 0.942		
Honey use before age one						
Yes	111	12.2	19.16 (1.45)	16–21	12.62 (1.07)	11–14
No	796	87.8	30.81 (6.55)	20–47	22.53 (5.54)	11–39
	T = 18.65, p < 0.001			T = 18.78, p < 0.001		
Knowledge of botulism						
Yes	39	4.3	32.66 (8.91)	20–47	28.28 (10.29)	13–39
No	868	95.7	29.24 (7.14)	16–46	21.00 (5.69)	11–38
	T = 2.89, p < 0.005			T = 7.45, p < 0.001		
Use of honey for health issues						
Yes	785	86.5	28.34 (7.14)	16–46	19.71 (4.73)	11–28
No	122	13.5	36.12 (3.24)	27–47	31.63 (3.49)	28–39
	T = 11.83, p < 0.001			T = 26.67, p < 0.001		
Use of honey for cough						
Yes	754	83.1	28.05 (7.09)	16–46	19.42 (4.60)	11–28
No	153	16.9	35.99 (3.40)	27–47	30.66 (3.69)	26–39
	T = 13.53, p < 0.001			T = 28.37, p < 0.001		
Use of honey for diarrhea						
Yes	94	10.4	19.93 (4.01)	16–31	12.12 (0.69)	11–13
No	813	89.6	30.48 (6.73)	20–47	22.38 (5.57)	14–39
	T = 14.88, p < 0.001			T = 17.82, p < 0.001		
Use of honey for oral mucositis						
Yes	127	14.0	20.11 (3.47)	16–31	12.61 (1.01)	11–14
No	780	86.0	30.90 (6.55)	21–47	22.73 (5.40)	14–39
	T = 18.13, p < 0.001			T = 21.02, p < 0.001		

There was a significant statistical difference in the mean THLS-32 scores of parents based on various factors such as parents age group (F = 19.54, p < 0.001), education level (F = 9.40, p < 0.001), honey use choice (F = 46.91, p < 0.001), knowledge of honey content (T = 16.21, p < 0.001), use of honey for children under one year (T = 18.65, p < 0.001), knowledge of botulism (T = 2.89, p < 0.001).

A statistically significant relationship has been identified between parents’ mean THLS-32 scores and the variables of using honey to treat their children’s health issues (T = 11.83, p < 0.001), alleviate cough (T = 13.53, p < 0.001), ameliorate diarrhea (T = 14.88, p < 0.001), and treat mucositis (T = 18.13, p < 0.001). No statistically significant differences were observed concerning child age and gender, parent gender, household income, as well as the source of obtaining honey and sources of information. Statistically significant differences were observed in the mean HCAMQ scores with regard to parents age group (F = 18.30, p < 0.001), an education level (F = 21.48, p < 0.001), the choice of honey use (F = 30.51, p < 0.001), honey use before the age of one (T = 18.78, p < 0.001), knowledge of botulism (T = 7.45, p < 0.005). A statistically significant differences has been identified between parents’ mean THLS-32 scores and the variables of using honey to treat their children’s health issues (T = 26.67, p < 0.001), alleviate cough (T = 28.37, p < 0.001), ameliorate diarrhea (T = 17.82, p < 0.001), and treat mucositis (T = 21.02, p < 0.001). No statistically significant differences were determined in terms of child age and gender, parent gender, household income, source of obtaining honey, knowledge of the content of honey, and sources of information about honey (Table 1).

The HCAMQ and THLS-32 mean scores of the parents were 21.31 and 29.39, respectively, and 73.9% had a limited/insufficient health literacy level (Table 2). A moderate correlation was found between the parents’ THLS-32 and HCAMQ mean scores (r = 0.662, p < 0.001).

**Table 2:** Parents’ mean scores on the HCAMQ and the THLS-32

Scale	N (%)	Mean SD	Min–max
HCAMQ			
Total	907 (100.0)	21.31 (6.13)	11–39
Holistic health	907 (100.0)	10.91 (2.67)	5–19
Complementary alternative medicine	907 (100.0)	10.47 (3.74)	6–21
THLS-32			
Total	907 (100.0)	29.39 (7.25)	16–47
Insufficient	391 (43.1)	22.55 (2.49)	16–25
Limited	279 (30.8)	30.62 (1.61)	26–33
Sufficient	189 (20.8)	37.82 (2.29)	34–42
Perfect	48 (5.3)	44.75 (1.06)	43–47

HCAMQ, Holistic Complementary and Alternative Medicine Questionnaire; THL-32, Turkey Health Literacy Scale-32

## Discussion

In this study, 86.5% of parents have utilized honey as a CAM for their children’s health issues. This rate was 27% in the only other study available on the same topic [8]. The difference between the two studies may be due to the fact that the data on honey presented in that study were not specifically about the use of honey but were part of a larger study on CAM use for children, and the parents may have spontaneously mentioned using honey for their children. However, this study was directly and exclusively about honey use, which could have been the reason for this stunning result. Nearly half of the parents reported a preference for honey due to its easy accessibility, while approximately one-third believed it to be more effective than medication.“This result was consistent with the study of Kumar et al. (2011), which showed that honey was considered a traditional home remedy that was convenient, easily accessible, and appropriate if symptoms were not severe [8].

The majority of the parents in this study stated that they used honey to treat their children’s coughs. A cough is one of the most commonly observed symptoms among children, and honey is widely used to treat it [5, 8, 10, 11]. Many studies on the use of CAM in the treatment of chronic diseases such as asthma as well as minor ailments have shown that parents use honey mixed with linden, lemon, milk, and radish juice to relieve cough symptoms [8, 10, 11]. The result of this study was consistent with the use of honey by parents in many cultures as a traditional remedy for alleviating cough. In addition to the World Health Organization’s 2001 recommendation regarding the use of honey as an antitussive, a recent meta-analysis has provided compelling evidence that honey effectively reduces both the frequency and severity of coughing [13, 15]. In light of the current results, it can be interpreted that parents’ preference for using honey to alleviate cough symptoms may be supported.

One noteworthy outcome of this study was that around one in every ten parents used honey in the treatment of their children’s diarrhea. Diarrhea is one of the most common medical disorders for which CAM is used [11, 34]. The literature has shown that parents commonly utilize various CAM modalities such as potato, yoghurt, banana, mint-lemon, coffee, and cola to manage diarrhea in their children [35, 36]. The result of the present study regarding the use of honey for diarrhea may have arisen because the research questions did not include other traditional methods but only queried the use of honey. While a study suggested that honey can be used as an adjunct to an oral rehydration solution to facilitate rapid recovery from vomiting and diarrhea in children and to restore the water balance in the body, the researchers highlighted the need for further studies with larger samples [18]. This striking result regarding parents using honey to treat their children’s diarrhea may be related to parents acquiring this information through media or social media channels. Nevertheless, a statistically significant difference between the parents’ information sources and the use of CAM could not be found. In future studies, a comparison of the current findings on honey usage for diarrhea treatment can be conducted by gathering comprehensive data on factors including frequency, reason, methodology, and information sources related to the use of honey. This will enable a more thorough analysis and evaluation.

In this study, more than one-tenth of parents have utilized honey for the treatment of oral mucositis. It has been observed that honey is among the traditional remedies used by mothers to address oral thrush infection [9]. In addition, honey is traditionally administered orally to newborns during their initial feedings or to calm crying infants. Furthermore, honey has been proposed as a viable candidate for complementary therapy in the management of radio/chemotherapy-induced oral mucositis [3, 8, 17, 37]. Researchers have indicated that apitherapeutic honey is a cost-effective treatment alternative due to its high viscosity, ability to form a physical barrier, inhibition of bacterial proliferation as an enzyme catalyst, and its capacity to accelerate epithelialization and angiogenesis owing to its rich nutritional content [14, 16, 18]. However, it is imperative to note that honey used for treatment must meet sterilization and medical-grade standards [14, 16]. In this study, the majority of the parents stated that they bought honey produced under natural conditions. However, the quality and medical effects of honey described as natural by the parents were not evaluated in this study; only the parents’ statements were assessed. For this reason, it is not possible to provide a recommendation to parents about the use of honey for oral mucositis treatment.

Notwithstanding, it is essential to recognize that the oral use of honey could potentially pose a significant risk factor for botulism. The results of this study reveal that a considerable number of parents initiated honey usage before their children reached one year of age, and a substantial portion of parents demonstrated a lack of awareness regarding the association between honey consumption and botulism. The practice of giving honey to infants is widespread not only in Turkey but also in regions such as the Middle East, Germany, Norway, Spain, and Venezuela. Nevertheless, it is crucial to emphasize that honey consumption represents the sole preventable risk factor for infant botulism, as it serves as a well-established pathway for Clostridium botulinum spores [9, 38]. Research conducted by the Centers for Disease Control and Prevention revealed a total of 524 botulism cases worldwide, excluding the United States, between 1976 and 2006. Among these cases, 63 were directly attributed to honey consumption [39]. While it remains uncertain whether honey was involved in cases of unspecified botulism, it is imperative to refrain from giving honey to infants below one year of age, as it can lead to severe health complications [40, 41]. Both the Centers for Disease Control and Prevention and the Ministry of Health advise against feeding honey to infants under the age of one year [40, 41]. In the current study, it was observed that parents who administered honey to their children before the age of one exhibited lower health literacy levels compared to those who did not use honey. Furthermore, their attitudes towards CAM were more positive. These findings hold significance as the majority of parents lacked knowledge regarding botulism, held positive attitudes towards CAM, and were unaware of its associated risks. It is evident that there is a need to educate parents about botulism, a condition with severe and potentially life-threatening consequences.

The current findings indicate that parents who used honey to address their children’s health issues, including conditions such as cough, diarrhea, and oral mucositis, exhibited lower levels of health literacy but held more positive attitudes toward the utilization of CAM compared to those who did not use honey. Furthermore, among those who were informed about the content of honey, higher health literacy levels were observed, although their attitudes towards CAM remained similar. These outcomes suggest that parents commonly use honey as a culturally and traditionally rooted remedy, often without full awareness of its potential effects. Notably, Turkey ranks as the world’s second-largest honey producer [42]. While this extensive production enhances accessibility, it also reinforces the traditional usage of honey. It is important to highlight that this study was conducted in a rural area rather than a metropolitan city, and this rural setting likely contributed to the parents’ easy access to natural honey. Roughly two-thirds of the parents included in this study exhibited either limited or insufficient health literacy levels. Simultaneously, they displayed positive attitudes towards CAM. An essential finding of this study is the significant positive correlation identified between low health literacy and positive attitudes towards CAM. It’s worth noting that the existing body of literature on the connection between health literacy levels in adults and their CAM attitudes lacks uniformity, and no comparable study in the realm of child health was identified for comparison [21–24]. The utilization of CAM is connected with making personal healthcare decisions while considering a range of potential benefits and adverse effects [20]. Among the various factors related to making and implementing healthcare decisions and assessing their outcomes, an individual’s health literacy holds significance. Inadequate levels of health literacy may have an adverse effect on one’s ability to make informed health decisions and assess their consequences [20]. Consequently, it can be inferred from the results of this study that parents had limited abilities to assess CAM methods, suggesting a need for further support in this area. Strengthening health literacy can serve as a preventive measure to promote the appropriate and reliable use of CAM. Further research is warranted to validate the findings of this study and explore the association between parental health literacy and CAM utilization for pediatric health concerns.

Some limitations should be taken into account when interpreting the findings of this study. Firstly, inherent biases are associated with self-administered, anonymous survey research conducted in the Turkish context, particularly related to online recruitment. These biases encompass non-representative sampling, self-selection bias, and the exclusion of parents without internet access. Therefore, the results cannot be considered representative of the entire population of Turkish parents with children aged 0–72 months. Secondly, this study had a cross-sectional nature and did not investigate temporal relationships or causality. Thirdly, the analysis did not encompass the methods employed when using honey as a CAM. Additional analysis that includes these methods may have assisted in understanding the prevalence of honey usage in the population.

## Conclusion

Although previous research has demonstrated parental utilization of honey for treating children’s coughs, this study is probably the first, to the best of our current knowledge, to establish its usage rates for diarrhea and oral mucositis and to explore the association between parents’ health literacy and their use of CAM. A significant positive correlation was identified between low health literacy and a positive attitude toward CAM. The majority of parents displayed positive attitudes toward CAM and possessed insufficient or limited health literacy. Given the high rate of positive CAM attitudes among the parents, their utilization of honey can be considered a noteworthy observation. However, their low levels of health literacy can be perceived as a critical risk factor when assessing potential risks and harms to child health and endeavouring to prevent possible negative outcomes. Health professionals specializing in pediatrics should assess parents’ knowledge regarding the use of honey as one of the CAM therapies and educate them about its safe utilization in order to minimize potential accompanying risks.

## Data Availability

The datasets generated and/or analyzed during the study are available from the corresponding author on reasonable request.

## Abbreviations

CAM: Complementary and Alternative Medicine
HCAMQ: Holistic Complementary and Alternative Medicine Questionnaire
THLS-32: Turkey Health Literacy Scale-32
